# Coexpression of CD163 and CD141 identifies human circulating IL-10-producing dendritic cells (DC-10)

**DOI:** 10.1038/s41423-019-0218-0

**Published:** 2019-03-06

**Authors:** Michela Comi, Daniele Avancini, Francesca Santoni de Sio, Matteo Villa, Molly Javier Uyeda, Matteo Floris, Daniela Tomasoni, Alessandro Bulfone, Maria Grazia Roncarolo, Silvia Gregori

**Affiliations:** 1 San Raffaele Telethon Institute for Gene Therapy (SR-TIGET), San Raffaele Scientific Institute (IRCCS), Milan, Italy; 2 PhD Program in Translational and Molecular Medicine (DIMET), University of Milan-Bicocca, Milan, Italy; 3 Division of Stem Cell Transplantation and Regenerative Medicine, Department of Pediatrics, ISCBRM, Stanford School of Medicine, Stanford, CA USA; 4 CRS4, Technology Park Polaris, Pula, Italy

**Keywords:** Dendritic cells, IL-10, T regulatory type 1 (Tr1) cells, Tolerance, Dendritic cells, Immune tolerance, Immune tolerance

## Abstract

Tolerogenic dendritic cells (DCs) are key players in maintaining immunological homeostasis, dampening immune responses, and promoting tolerance. DC-10, a tolerogenic population of human IL-10-producing DCs characterized by the expression of HLA-G and ILT4, play a pivotal role in promoting tolerance via T regulatory type 1 (Tr1) cells. Thus far, the absence of markers that uniquely identify DC-10 has limited in vivo studies. By in vitro gene expression profiling of differentiated human DCs, we identified CD141 and CD163 as surface markers for DC-10. The coexpression of CD141 and CD163 in combination with CD14 and CD16 enables the ex vivo isolation of DC-10 from the peripheral blood. CD14^+^CD16^+^CD141^+^CD163^+^ cells isolated from the peripheral blood of healthy subjects (ex vivo DC-10) produced spontaneously and upon activation of IL-10 and limited levels of IL-12. Moreover, in vitro stimulation of allogeneic naive CD4^+^ T cells with ex vivo DC-10 induced the differentiation of alloantigen-specific CD49b^+^LAG-3^+^ Tr1 cells. Finally, ex vivo DC-10 and in vitro generated DC-10 exhibited a similar transcriptional profile, which are characterized by an anti-inflammatory and pro-tolerogenic signature. These results provide new insights into the phenotype and molecular signature of DC-10 and highlight the tolerogenic properties of circulating DC-10. These findings open the opportunity to track DC-10 in vivo and to define their role in physiological and pathological settings.

## Introduction

Dendritic cells (DCs) are regulators of innate and adaptive immune responses. Different DC subsets drive these responses towards immunity or tolerance. In human blood, two major DC subtypes have been identified as follows: conventional/classical (c)DCs and plasmacytoid (p)DCs.^1^ Activated cDCs secrete IL-12 and TNF-α and prime naïve CD4^+^ and CD8^+^ T cells.^2,3^ In steady-state conditions, circulating cDCs have an immature phenotype, maintain immunological homeostasis, and can induce tolerance. pDCs, upon recognition of foreign nucleic acids, produce type I interferons (IFNs) and acquire the capacity to present foreign antigens (Ags). pDCs express lower levels of costimulatory and MHC class II molecules as compared to cDCs and promote T regulatory (Tregs)^4–6^. In addition to immature cDCs, different subsets of DCs, termed tolerogenic (tol)DCs, play a role in promoting tolerance, thus acting as regulatory cells.^7,8^ TolDCs can be induced by immunosuppressive compounds, anti-inflammatory cytokines, or genetic modifications.^9,10^ The regulatory capacity of tolDCs depends on the expression of low levels of costimulatory molecules, expression of inhibitory and/or modulatory receptors, secretion of low amounts of pro-inflammatory cytokines, and high level secretion of anti-inflammatory cytokines. These characteristics lead to inhibition of effector T cell responses and induction of Tregs.^11^

We identified and characterized a subset of human DCs, termed DC-10, because of their ability to spontaneously secrete IL-10. DC-10 can be differentiated in vitro by culturing peripheral monocytes in the presence of exogenous IL-10.^12^ DC-10 express CD11c, CD14, and CD16, have a mature phenotype (i.e., express CD83 and CD86), and produce IL-10 in the absence of IL-12, a feature that, together with HLA-G and immunoglobulin-like transcript (ILT)-4 expression, is required for DC-10 mediated induction of T regulatory type 1 (Tr1) cells.^12,13^ In vitro differentiated DC-10 are currently used to generate Tr1 cells suitable for cell-based therapies.^14–16^ DC-10 were identified in the peripheral blood and spleen of healthy donors^12^ and in the decidua of pregnant women^17^ as CD11c^+^CD14^+^CD83^+^ cells; however, these markers are not ubiquitously expressed by DC-10, and CD83 expression is influenced by activation.

In this study, we investigated the gene expression profile of in vitro differentiated DC-10 to search for specific surface markers allowing for the unequivocal in vivo identification of DC-10. We show that CD141 and CD163 are highly and stably expressed by DC-10 and that the expression of these markers, in combination with CD14 and CD16, allows for the identification and isolation of DC-10 from the peripheral blood and spleen of healthy subjects. Ex vivo isolated DC-10 induce Tr1 cell differentiation in vitro and show a transcriptional profile similar to that of in vitro differentiated DC-10.

## Methods

### Cell preparation

Human peripheral blood was obtained from healthy donors in accordance with local committee approval (PERIBLOOD and TIGET09) and with the Declaration of Helsinki. Peripheral blood mononuclear cells (PBMCs) were isolated by density gradient centrifugation over Lymphoprep^TM^ (Axis-Shield PoC AS, Norway). The monocyte fraction of PBMCs was enriched by a Percoll gradient (GE Healthcare, USA), as previously described.^18^ Human spleens were obtained from cadaveric donors through the North Italian Transplant Organization upon informed consent from a first-degree relative in accordance with the local ethical committee approval and with the Declaration of Helsinki. Spleen cells were obtained by mechanical disruption of the organ followed by density gradient centrifugation over a Lymphoprep^TM^ gradient.

### DC differentiation

CD14^+^ cells were isolated from PBMCs by positive selection using CD14 MicroBeads (Miltenyi Biotech, Germany) according to the manufacturer’s instructions. Cells were cultured in RPMI 1640 medium (Lonza, Switzerland) with 10% fetal bovine serum (FBS) (Euroclone, Italy), 100 U/ml penicillin/streptomycin (Lonza, Switzerland), and 2 mM l-glutamine (Lonza, Switzerland), at 10^6^ cells/ml at a 1 ml volume in a 24-well culture plate, supplemented with rhGM-CSF (Miltenyi Biotech, Germany) at 100 ng/ml and rhIL-4 (Miltenyi Biotech, Germany) at 10 ng/ml for 7 days at 37 °C with 5% CO_2_. One milliliter per well of fresh prewarmed medium with cytokines, at concentrations as stated above, was added on day 3. To obtain mature (m)DCs, cells were activated on day 5 by the addition of 1 μg/ml of LPS (Sigma Aldrich, CA, USA) for an additional 2 days, while immature (i)DCs were left unstimulated. DC-10 were generated as above, with the addition of rhIL-10 (CellGenix, Germany) at 10 ng/ml at days 0, 3, and 5. In some experiments, 1 μg/ml of LPS (Sigma Aldrich, CA, USA) was added at day 5 during DC-10 differentiation to generate DC-10+LPS cells or cells were stimulated with TLR2/3/5/9 agonists (InvivoGen, CA, USA) using 10^8^ cells/ml of Heat Killed Listeria Monogenic (HKLM), 10 μg/ml of Poli (I:C), 1 μg/ml of Flagellin *S. typhimurium*, or 5 μM of CpG ODN 2006. In vitro differentiated DCs were harvested on day 7 for phenotypical, molecular, and functional analyses.

### Flow cytometry and sorting

Fluorochrome-conjugated antibodies against the following Ags were used for DC staining in PBMCs and spleen: CD1a, CD14, CD16, HLA-DR, CD11c, CD35, FPR1, CD163 (Becton Dickinson, CA, USA), CD141 (Miltenyi Biotech, Germany), CLEC4G (R&D system, MN, USA), CD45, and ENG (Biolegend, CA, USA). A two-step staining was performed for KITLG (Abcam, OR, USA) and LILRB5 (R&D system, USA) with a donkey anti-rabbit (Biolegend, CA, USA) and anti-goat (R&D system, USA) secondary antibody, respectively.

The following fluorochrome-conjugated antibodies were used for CD4^+^ T cell staining: anti-CD3, anti-CD45RA, anti-CD25, anti-HLA-DR (Becton Dickinson, CA, USA), anti-CD4 (Biolegend, CA, USA), anti-CD49b, and anti-LAG-3 (Miltenyi Biotec, Germany). For Tr1 cell detection, CD4^+^ T cells were stained as previously described.^19^

FcR Blocking Reagent (Miltenyi Biotech, Germany) was used in all preparations to avoid nonspecific staining. In brief, cells were centrifuged and resuspended in Dulbecco’s Phosphate-Buffered Saline (DPBS, Corning) supplemented with 2% FBS (Lonza, Switzerland). Cells were incubated at room temperature for 15 min, centrifuged and fixed with 1% formaldehyde methanol-free solution (Thermo Fisher Scientific, MA, USA). For cultured cells, the described passages were preceded by staining with the LIVE/DEAD^TM^ Fixable Dead Cell Stain Kit (Invitrogen, CA, USA) following the manufacturer’s instructions.

For Ki67 staining, after surface staining with anti-CD4 and anti-CD3, cells were fixed, permeabilized, and stained with anti-Ki67 (Becton Dickinson, CA, USA) using Foxp3/Transcription Factor Staining Buffer Set (eBioscience, USA). Samples were acquired using the FACSCanto II or Fortessa Flow Cytometers (Becton Dickinson, CA, USA), and data were analyzed with FlowJo software (FlowJo, LLC, USA).

For sorting, the Percoll-enriched monocyte fraction of PBMCs was resuspended in DPBS with 2% FBS and 2 mM EDTA (Lonza, Switzerland) and stained with anti-CD1c, anti-CD141 (Miltenyi Biotech, Germany), anti-CD11c, anti-CD163, anti-CD16 (Becton Dickinson, CA, USA), and anti-CD14 (Biolegend, CA, USA) antibodies and FcR Blocking Reagent (Miltenyi Biotec, Germany) for 15 min. Cells were then washed and resuspended in DPBS with 2% FBS and 2 mM EDTA and sorted using the FACSAria II or FACSAria Fusion Cell Sorters (Becton Dickinson, CA, USA) with a 100-μm nozzle. Phenotypical, molecular, and functional analyses were performed on ex vivo isolated cells.

Cells analyzed without any manipulation (directly after isolation) are referred to as “in vivo” cells, while cells that underwent sorting and were then cultured in vitro are referred to as “ex vivo” cells.

### Microarray

Total RNA was isolated using an RNeasy Kit (QIAGEN, CA, USA) according to the manufacturer’s instructions. Preparation of terminal-labeled complementary DNA (cDNA), hybridization to the whole-transcript GeneChip Human Gene 1.0 ST Array (Affymetrix, USA) and scanning of the arrays were performed according to the manufacturer’s protocols. Raw data were preprocessed with the robust multichip average (RMA) algorithm. Genes were considered differentially expressed if log_2_ fold change values were >1 or <−1, with *P* < 0.05. All these steps were conducted using R packages *affy* and *limma* hosted on the Bioconductor website.^20,21^

### RT-PCR

Total RNA was extracted using an RNeasy Kit (QIAGEN, CA, USA), and cDNA was synthesized with a high-capacity cDNA Reverse Transcription Kit (Applied Biosystems, CA, USA) according to the manufacturer’s instructions. cDNA from mDCs, iDCs, and DC-10 was loaded in Low Density TaqMan® cards with TaqMan Universal PCR Master Mix (Applied Biosystems, CA, USA), and PCR was performed on an ABI Prism 7900 HT Sequence Detection System (Applied Biosystems, CA, USA) following the manufacturer’s instructions. SDS 2.2.1 software was used to analyze the data, using RPL0 as an endogenous control. Quantification relative to the endogenous control was carried out using the following formula: ∆CT = CT_gene_-CT_RPL0_; relative gene expression = 2^−∆CT^.

### T cell isolation and proliferation

CD4^+^ T cells were purified from PBMCs by negative selection using the human CD4^+^ T cell Isolation Kit II (Miltenyi Biotech, Germany) according to the manufacturer’s instructions. CD4^+^ T cells were then depleted of CD45RO^+^ cells using anti-CD45RO microbeads (Miltenyi Biotech, Germany). CD4^+^CD45RO^−^CD45RA^+^ T cells were consistently >90% of purified products. CD4^+^CD45RO^−^ T cells were labeled with Cell Proliferation Dye eFluor® 670 (eBioscience, CA, USA) according to the manufacturer’s instructions and stimulated with 10^4^ allogeneic sorted DC-10 (ex vivo DC-10) or conventional DCs (ex vivo cDCs) (10:1, T:DCs) in X-VIVO 15 medium (Lonza, Switzerland) supplemented with 5% human serum (Sigma Aldrich, CA, USA) and 100 U/ml penicillin/streptomycin (Lonza, Switzerland). After 5 days, T cells were collected and washed, and their phenotype and proliferation were analyzed by flow cytometry.

### T cell differentiation and suppression assay

CD4^+^CD45RO^−^ T cells (1×10^6^) were cultured with 5×10^4^ allogeneic sorted DC-10 (ex vivo DC-10) or conventional DCs (ex vivo cDCs) (20:1, T:DCs) in X-VIVO 15 medium (Lonza, Switzerland) supplemented with 5% human serum (Sigma Aldrich, CA, USA) and 100 U/ml penicillin/streptomycin (Lonza, Switzerland). After 10 days, primed T cells were collected, washed, and analyzed. T cells stimulated with DC-10 are referred to as T(DC-10) cells, while those stimulated with cDCs are referred to as T(cDC) cells.

T(DC-10) and T(cDC) cells were plated with in vitro differentiated iDCs that were autologous to ex vivo DC-10 and ex vivo cDCs (10:1, T:DCs), and in some experiments, 100 U/ml of IL-2 (Chiron, Italy) was exogenously added to T(DC-10) cell cultures. As a control, T(DC-10) and T(cDC) cells were stimulated with Dynabeads^TM^ Human T-Activator CD3/CD28 (5:1, cells:beads). After 3 days of stimulation, T cells were collected and washed, and cell proliferation was evaluated by Ki67 staining via flow cytometry.

To evaluate the suppressive activity of T(DC-10) cells, T(cDC) cells (responder cells) were stained with Cell Proliferation Dye eFluor® 450 (eBioscience, CA, USA) and activated with iDCs autologous to ex vivo DC-10 and ex vivo cDCs in the presence of T(DC-10) cells at a 1:1 ratio (totalT:iDCs ratio was 10:1). After 3 days, the percentages of divided responder T cells were calculated by proliferation dye dilution by flow cytometry.

### Cytokine determination

A total of 10^5^ FACS-sorted cells were plated in at a 100 μl final volume. Cells were left un-stimulated or activated with 200 ng/ml LPS (Sigma, CA, USA), and supernatants were collected after 48 h. Levels of IL-6, IL-10, IL-12, and TNF-α were determined by a 4-plex Bio-Plex system according to the manufacturer’s instructions (Bio-Rad, CA, USA). The production of IFN-γ and GM-CSF by CD4^+^ T cells was quantified in coculture supernatants by a BD OptEIA^TM^ ELISA Kit (Becton Dickinson, CA, USA).

### RNA-Seq analysis and data processing

RNA was isolated using an RNeasy Micro or RNA MiRNeasy Kit (QIAGEN, CA, USA), and 10 ng of total or poly-adenylated RNA was converted to cDNA by random primer amplification. The library was constructed and amplified from cDNA using the Nextera DNA Library Prep Kit with unique dual adapters (Illumina, NY, USA). Samples were multiplexed and sequenced to 1.5–2.0 × 10^7^, 2 × 150-bp reads per sample on a NextSeq500 (Illumina, NY, USA). Raw RNA sequences (≥3×10^7^ per sample) were trimmed using Skewer and then aligned against the human reference genome *GRChv38* using STAR (v2.5.3a).^22,23^ DESeq2 was used to determine the total counts per gene for transcripts and to calculate differential gene expression between samples and clustering.^24^ Gene lists of the 500 most significantly up‐ and down-regulated genes in in vitro DC-10 compared to mDCs were tested for enrichment in ex vivo DC-10 and cDC transcriptomes by the Broad Institute’s Gene Set Enrichment Analysis tool.^25^ To find significantly enriched biological processes, differentially expressed genes (DEGs) were analyzed using the Enrichr online tool,^26^ and the results were visualized using REVIGO.^27^

### Statistical analysis

Wilcoxon matched pairs test (two-tailed) was used for statistical analysis. For microarray and RNAseq data, hierarchical clustering was applied, and adjusted *P*-values (Benjamini-Hochberg) were used to identify significant DEGs. All results are presented as the mean values±standard deviations. Differences were regarded as significant at *P < 0.05, **P < 0.01, ***P < 0.001 and ****P < 0.0001. The results were analyzed using GraphPad Prism 5.0 (GraphPad Software, CA, USA).

### GEO accession numbers

Transcriptome data are available at the GEO repository under the following accession numbers: GSE117947 (Superseries), GSE117945 (RNA-seq), and GSE117946 (MicroArray).

## Results

### DC-10 are a transcriptionally stable and unique human DC subset

**Fig. 1:**
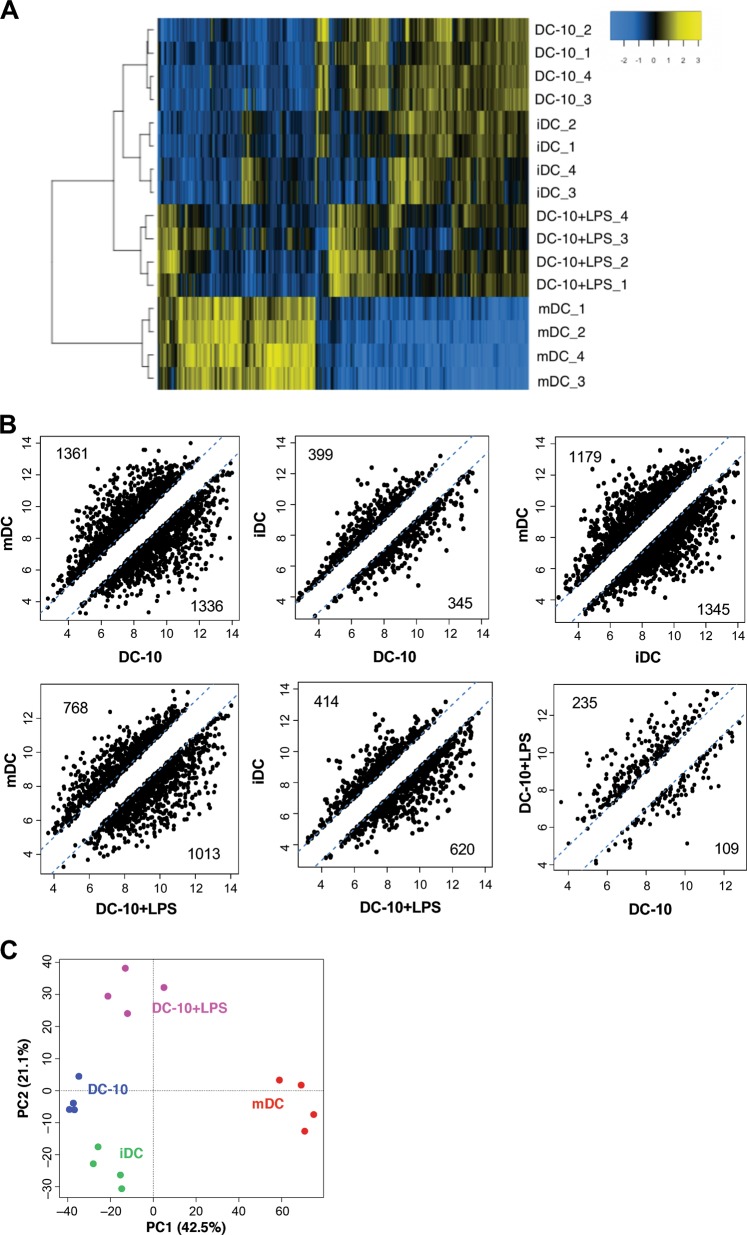
Transcriptional profile of in vitro differentiated DC-10. Mature (m)DCs, immature (i)DCs, DC-10, and DC-10 activated with LPS (DC-10+LPS) were differentiated in vitro from peripheral blood monocytes of healthy donors (*n* = 4) according to the Methods section, and microarray analysis was performed. **a** Two-dimensional heatmap of significant differentially expressed genes (DEGs) (*P* < 0.05, absolute fold change (FC) value >1). The color scale is relative to the mean expression levels. Genes are in columns, and samples are in rows. Data in rows have been hierarchically clustered. **b** Scatterplots of differential expression between mDCs, iDCs, DC-10, and DC-10+LPS. Each dot represents the averaged Log_2_ FC value of a single gene. Dashed diagonal lines indicate the thresholds for FC (absolute FC value >1). Numbers indicate DEGs. **c** Principal component analysis plot for expression data in mDCs, iDCs, DC-10, and DC-10+LPS

To define the gene expression profile of DC-10, we compared their transcriptome with that of immature (i)DCs and mature (m)DCs differentiated from peripheral blood monocytes of the same healthy donors. To evaluate the stability of the DC-10 transcriptome, we included DC-10 activated with LPS (DC-10+LPS) in the analysis. This stimulation was chosen as one representative stimulus among different TLR agonists tested since stimulation with all agonists resulted in a similar maintenance of CD14, HLA-DR, and CD86 expression levels in DC-10 (Figure S1), and LPS was the stimulus used to generate mDCs. The expression profile of DC-10 was consistent among different donors, stable upon LPS activation, and similar to that of iDCs, which are recognized as tolerogenic cells,^28,29^ and is distinct from that of mDCs (Fig. 1a). The high expression of *CD14*, *FCGR3A* (encoding for CD16), *LILRB2* (encoding for ILT4), and *IL10* and the low expression of *lL12B* (encoding for IL-12p40) were verified in the microarray, confirming the quality of the dataset (Figure S2). By setting log_2_ fold change (FC) and *P-*value cut offs at −1/1 and 0.05, respectively, we identified 2697 and 744 DEGs in DC-10 compared to mDCs and iDCs, respectively (Fig. 1b). The comparison between the transcriptome profiles of DC-10 and DC-10+LPS revealed the lowest number of DEGs (344), indicating that DC-10 have a stable transcriptional profiles (Fig. 1b). Moreover, differences in the transcriptome profile of DC-10 compared to mDCs and iDCs were maintained upon LPS activation (with 1781 and 1034 DEGs, respectively, Fig. 1b). Principal component analysis summarized the observed transcriptional similarity among DC-10, DC-10 + LPS, and iDCs and the divergence from mDCs (Fig. 1c). These results demonstrate that in vitro differentiated DC-10 have a defined transcriptional profile that is stable upon TLR stimulation.

**Fig. 2:**
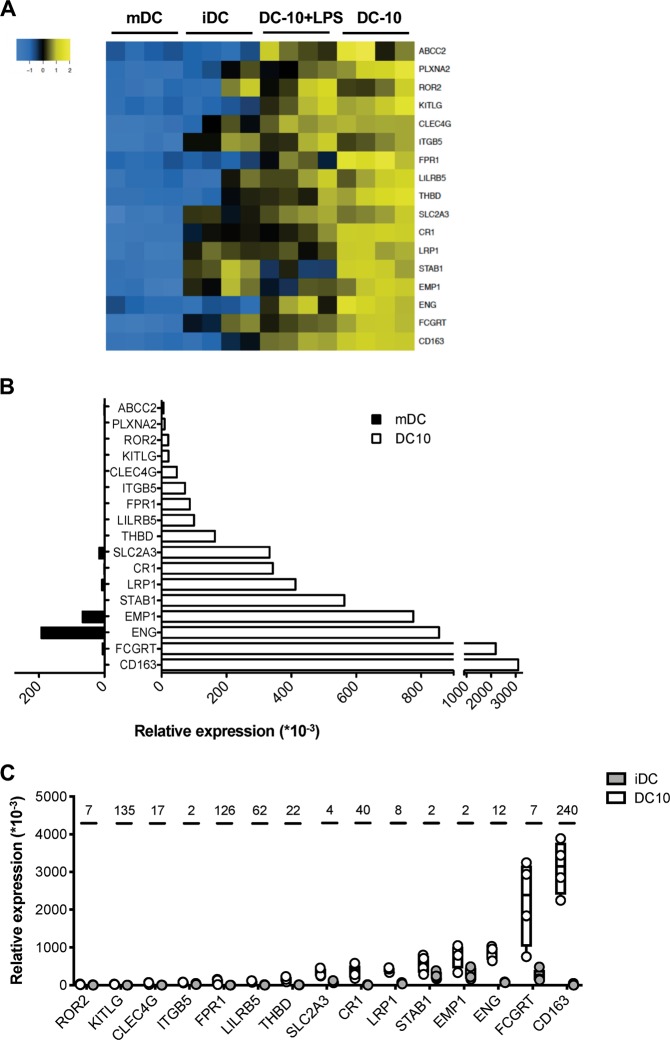
Validation and selection of putative DC-10 markers. **a** Two-dimensional heatmap of filtered differentially expressed genes (DEGs) encoding candidate DC-10 markers is shown. The color scale is relative to the mean expressional levels. Genes are in rows, and the samples are in columns. **b**, **c** DC-10, mDCs, and iDCs were differentiated in vitro from peripheral blood monocytes of additional healthy donors (*n* = 4), and RT-PCR was performed. Relative mRNA expression of the indicated genes in DC-10, mDCs, and iDCs are normalized to housekeeping gene RPL0 and is shown using numbers representing arbitrary units. The average relative mRNA expression of the indicated genes in mDCs (black) and DC-10 (white) is presented **b**. Relative mRNA expression of the indicated genes in DC-10 and iDCs is shown; each dot represents a single donor, lines indicate medians, and whiskers are minimum and maximum levels. Numbers indicate fold change values (**c**)

To identify specific markers of DC-10, we focused the analysis on DEGs encoding for cell surface proteins. We selected 17 genes significantly (*P* < 0.01) up-regulated in DC-10, at a steady state and upon LPS activation, compared to mDCs (Fig. 2a). RT-PCR on DC-10 and mDCs from four additional donors confirmed a higher expression of the selected genes in DC-10 compared to mDCs, with the exception of *PLXNA2* and *ABCC2* (Fig. 2b). To assess the specificity of the 15 validated genes, we analyzed their expression in iDCs, and results showed higher expression of 13 out of 15 genes in DC-10 compared to iDCs (Fig. 2c).

**Fig. 3:**
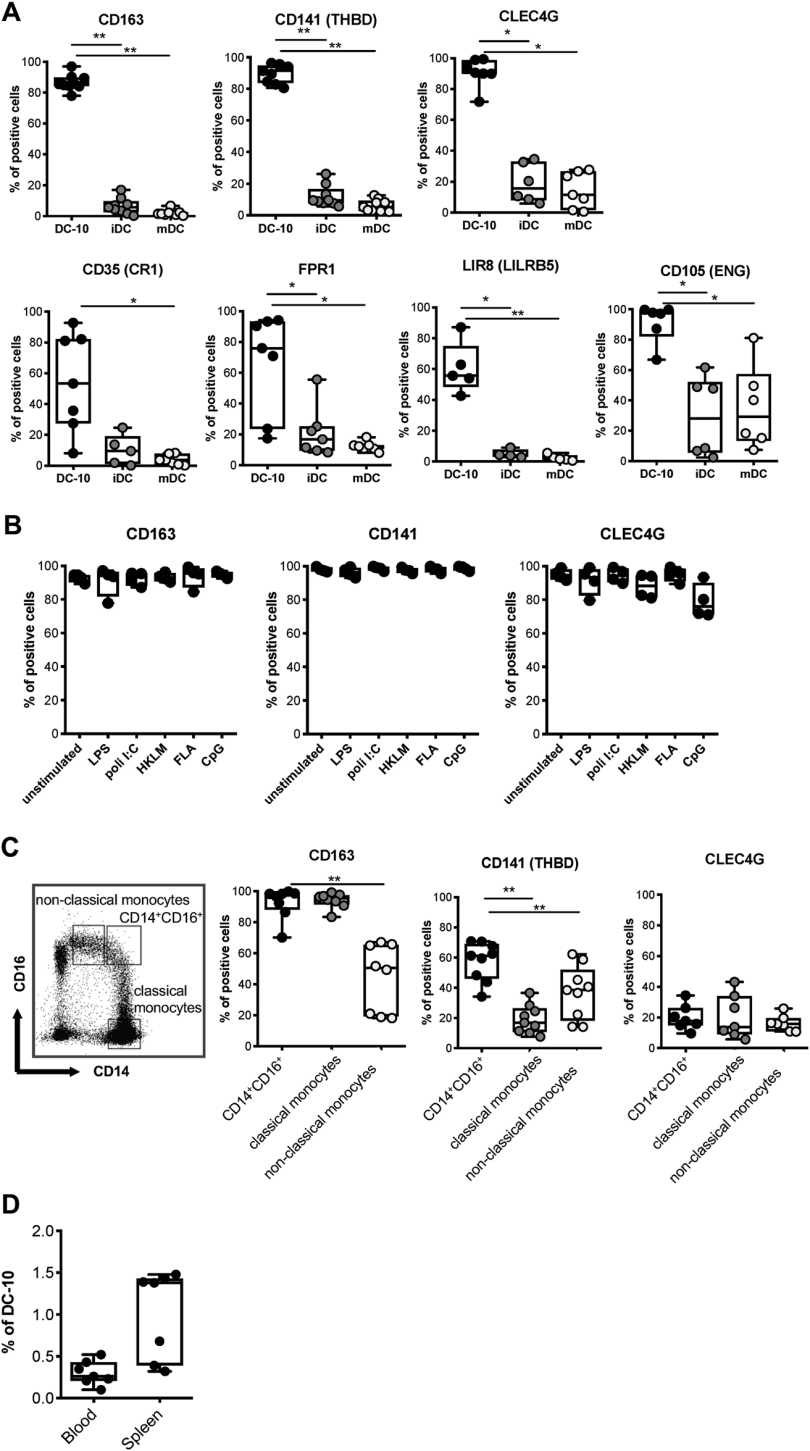
Co-expression of CD14, CD16, CD141, and CD163 identifies in vitro differentiated DC-10 and putative DC-10 in vivo. **a** DC-10, mDCs, and iDCs were differentiated in vitro from peripheral blood monocytes of healthy donors (*n* = 5–7) according to the Methods section. Expression levels of the indicated markers were measured by flow cytometry. The following gating strategy was applied: doublet exclusion, followed by alive cells and gating on CD11c^+^ cells. Each dot represents a single donor, lines indicate medians, and whiskers are minimum and maximum levels. **b** Expression levels of CD141, CD163, and CLEC4G were evaluated in DC-10 activated with the indicated TLRs at day 5 of differentiation (*n* = 4). The following gating strategy was applied: doublet exclusion, followed by alive cells and gating on CD11c^+^ cells. Each dot represents a single donor, lines indicate medians, and whiskers are minimum and maximum levels. **c** Expression levels of CD141, CD163, and CLEC4G were evaluated on CD14^+^CD16^+^ cells that contain DC-10, on CD14^+^CD16^−^ (classical), and on CD14^low^CD16^+^ (non-classical) monocytes (*n* = 7–9). The following gating strategy was applied: doublet exclusion, followed by alive cells and indicated gating. Each dot represents a single donor, lines indicate medians, and whiskers are minimum and maximum levels. **d** CD14^+^CD16^+^CD141^+^CD163^+^ cell frequencies in peripheral blood of healthy donors and in splenic CD45^+^ cells of cadaveric donors are shown (*n* = 7). Each dot represents a single donor, lines indicate medians, and whiskers are minimum and maximum levels. *P ≤ 0.05, **P ≤ 0.01 (Wilcoxon matched pairs test, two-tailed)

We investigated the protein expression levels of the genes showing at least 10 times higher mean fold change transcript expression in DC-10 compared to iDCs in in vitro differentiated additional donors. Flow cytometric data showed that DC-10 expressed significantly higher levels of CD163 (*P* = 0.039), CD141 (encoded by *THBD, P* = 0.039), and CLEC4G (*P* = 0.013) compared to both iDCs and mDCs (Fig. 3a, upper panel, and Figure S3). The expression of CD35 (encoded by *CR1*), FPR1, and LIR8 (encoded by *LILRB5*) in DC-10 was significantly higher compared to mDCs. FPR1 and LIR8 expression was higher in DC-10 compared to iDCs; however, the expression of the above markers was low in DC-10 and varied among the donors investigated (Fig. 3a, lower panels, and Figure S3). Finally, CD105 (encoded by *ENG*) was highly expressed on DC-10 in all donors tested, but it was also expressed at variable levels in iDCs and mDCs, with CD105 expression nearly comparable to that of DC-10 in one donor (Fig. 3a, lower panels, and Figure S3). We selected CD141, CD163, and CLEC4G and analyzed their expression on DC-10 upon activation with different TLR agonists. The results in Fig. 3b demonstrate that CD141, CD163, and CLEC4G are firmly expressed on DC-10 independently of TLR-mediated activation, indicating that CD141, CD163, and CLEC4G are selectively and stably expressed by in vitro differentiated DC-10.

### Co-expression of CD141 and CD163 in combination with CD14 and CD16 identifies functional DC-10 in vivo

We analyzed the expression of CD141, CD163, and CLEC4G on peripheral blood CD14^+^CD16^+^ cells, which contain DC-10.^12^ CD14^+^CD16^−^ (classical) and CD14^low^CD16^+^ (non-classical) monocytes served as controls. The vast majority of CD14^+^CD16^+^ cells and classical monocytes were CD163^+^, while a significantly (*P* = 0.008) lower frequency of non-classical monocytes expressed CD163. Within CD14^+^CD16^+^ cells, a significantly (*P* = 0.004) higher proportion of cells expressed CD141 compared to both classical and non-classical monocytes. Conversely, CLEC4G was expressed at low levels in all populations (Fig. 3c and S4). Thus, CD163 expression can be used to discriminate CD14^+^CD16^+^ DC-10 from non-classical monocytes, whereas coexpression of CD16 and CD141 segregates CD14^+^CD16^+^ DC-10 from classical monocytes. In accordance with our previous study,^12^ CD14^+^CD16^+^CD141^+^CD163^+^ DC-10 represent 0.3 ± 0.14% (*n* = 7) of total PBMCs and 1.01 ± 0.52% (*n* = 7) of CD45^+^ cells in the spleen of healthy subjects (Fig. 3d).

**Fig. 4:**
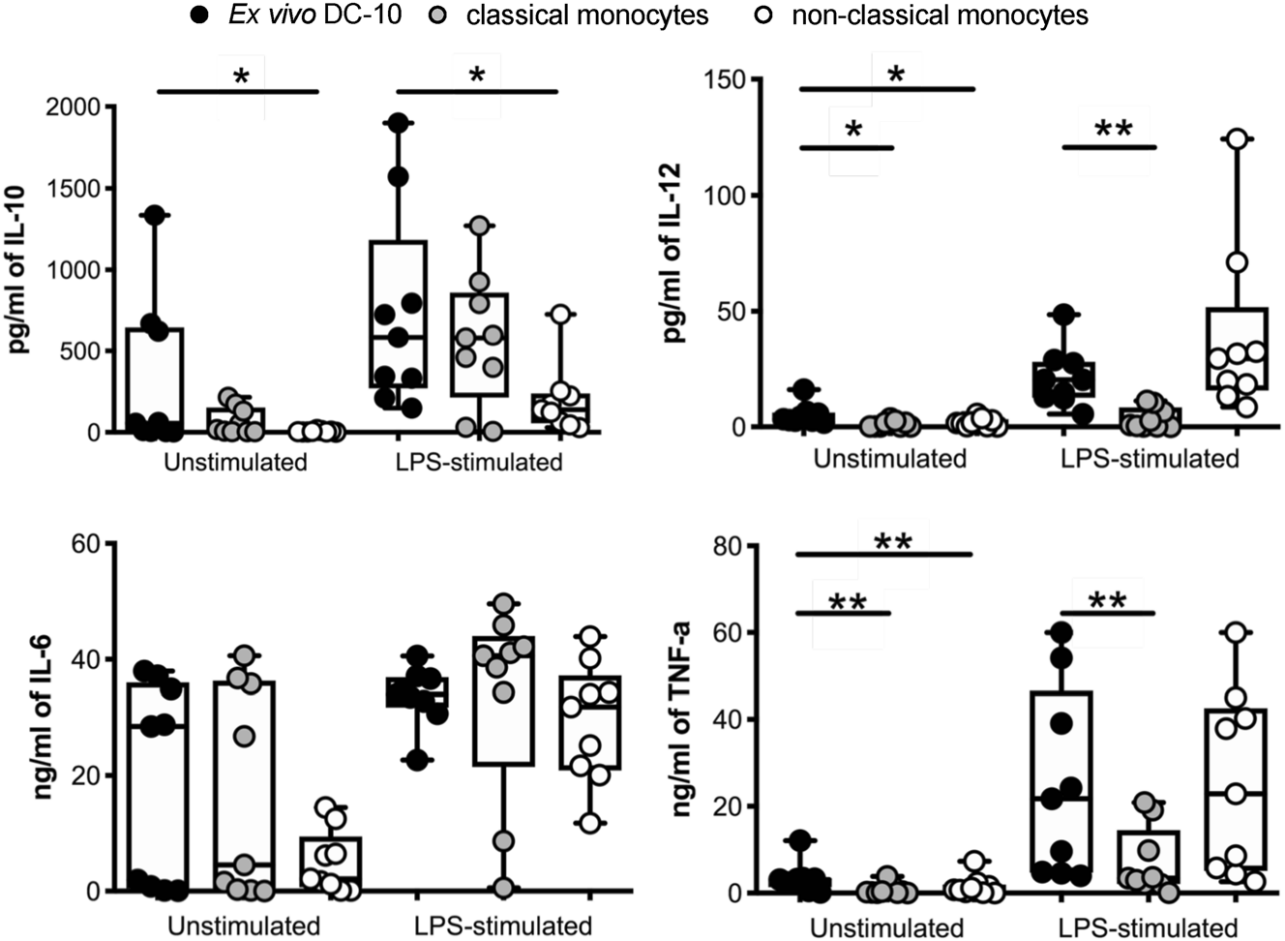
Ex vivo DC-10 secrete IL-10 spontaneously and upon activation. Ex vivo DC-10 (CD14^+^CD16^+^CD141^+^CD163^+^), classical (CD14^+^CD16^−^) and non-classical (CD14^+^CD16^+^CD163^−^) monocytes were FACS-isolated from the peripheral blood of healthy donors and left unstimulated or stimulated with LPS for 48 h (*n* = 9). Concentration levels of IL-10, IL-12, TNF-α, and IL-6 in culture supernatants were evaluated by a multiplex microbead-based cytokine array. Each dot represents a single donor, lines indicate medians, and whiskers are minimum and maximum levels. *P ≤ 0.05, **P ≤ 0.01 (Wilcoxon matched pairs test, two-tailed)

We next assessed the cytokine profile of CD14^+^CD16^+^CD141^+^CD163^+^ (ex vivo DC-10) isolated by fluorescence-activated cell sorting (FACS) from healthy donors’ PBMCs (Figure S5). As controls, we isolated CD14^+^CD16^−^ (classical monocytes) and CD14^+^CD16^+^CD163^−^ (non-classical monocytes) cells. In unstimulated conditions, we observed high variability in cytokine production among donors tested, especially for IL-10 and IL-6, likely due to different activation levels of in vivo circulating cells. In some donors, ex vivo DC-10 produced high levels of IL-10 already at steady state, whereas in others they did not. Importantly, upon LPS stimulation, ex vivo DC-10 from all donors tested secreted increased amounts of IL-10, which were similar to those produced by activated classical monocytes but higher compared to non-classical monocytes (Fig. 4). All subsets secreted very low levels of IL-12 at a steady state and <80 pg/ml upon activation. In some donors, similar to classical monocytes, ex vivo DC-10 spontaneously produced higher levels of IL-6 compared to those produced by non-classical monocytes, while upon activation, these cells increased IL-6 secretion overall. Finally, at a steady state, all subsets secreted low levels of TNF-α that increased upon LPS activation, with ex vivo DC-10 producing significantly higher levels than those of classical monocytes but comparable to those of non-classical monocytes. Overall, these data indicate that ex vivo isolated CD14^+^CD16^+^CD141^+^CD163^+^ cells display a cytokine profile similar to that of in vitro differentiated DC-10,^12^ characterized by a high IL-10/IL-12 ratio.

### Ex vivo DC-10 promotes Tr1 cells in vitro

**Fig. 5:**
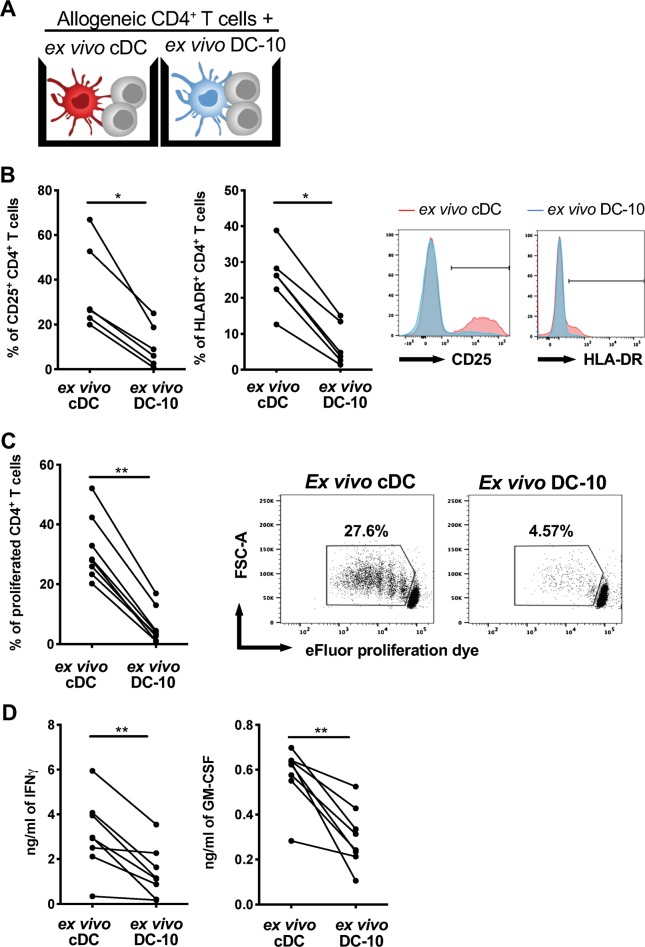
Ex vivo DC-10 promote hypo-responsiveness in allogeneic naïve CD4^+^ T cells. **a** Scheme of the experiment: naïve CD4^+^ T cells were cultured with ex vivo DC-10 (CD14^+^CD16^+^CD141^+^CD163^+^) or ex vivo cDCs (CD1c^+^CD11c^+^) FACS isolated from the peripheral blood of healthy donors (ratio 10:1) for 5 days. **b** Expression of the activation markers CD25 and HLA-DR on CD4^+^ T cells stimulated with ex vivo cDCs (red) or ex vivo DC-10 (blue) were evaluated by flow cytometry (*n* = 6). The following gating strategy was applied: doublet exclusion, followed by alive cells and gating on CD3^+^CD4^+^ cells. Left, each dot represents a single donor; right, flow cytometry histograms from one representative donor. **c** Naive CD4^+^ T cell proliferation was evaluated by proliferation dye dilution (*n* = 8). The following gating strategy was applied: doublet exclusion, followed by alive cells and gating on CD3^+^CD4^+^ cells. The percentage of proliferated cells is shown for each single donor (left panel) and in representative dot plots (right panels). **d** IFN-γ and GM-CSF in culture supernatants were measured by ELISA (*n* = 8). Each dot represents a single donor. *P ≤ 0.05 **P ≤ 0.01 (Wilcoxon matched pairs test, two-tailed)

Ex vivo DC-10 were evaluated for their effect on naïve CD4^+^ T cells. As a control, we used FACS-isolated CD1c^+^CD11c^+^ cells (ex vivo cDCs) that expressed HLA-DR, CD80, and CD86 at levels similar to those expressed by ex vivo DC-10 (Figure S6A-S6B). Cytokine profile of ex vivo cDC used is shown in Figure S6C. The ability of ex vivo DC-10 and ex vivo cDCs to stimulate allogeneic naïve CD4^+^ T cells was compared (Fig. 5a). The results showed that naïve CD4^+^ T cells cultured with ex vivo DC-10 expressed significantly (*P* = 0.03) lower levels of CD25 and HLA-DR, proliferated significantly (*P* = 0.008) less, and secreted significantly (*P* = 0.008) lower levels of IFN-γ and GM-CSF compared to T cells stimulated with cDCs (Fig. 5b, c). Thus, similar to in vitro differentiated DC-10, ex vivo DC-10 promote hypo-responsiveness in allogeneic CD4^+^ T cells.

**Fig. 6:**
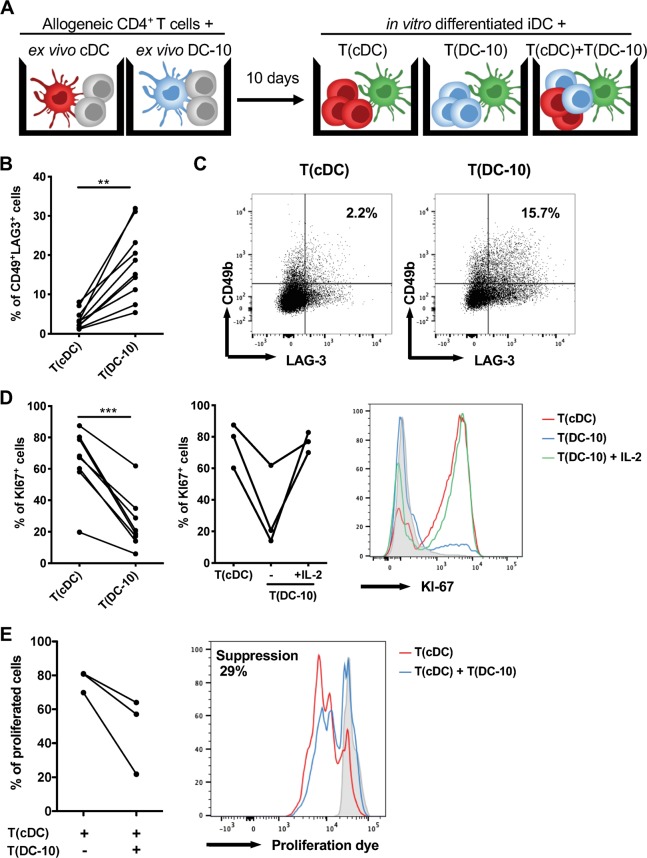
Ex vivo DC-10 induce anergic alloantigen-specific CD49^+^LAG-3^+^CD4^+^ T cells. **a** Scheme of the experiment: naïve CD4^+^ T cells were cultured with allogeneic ex vivo DC-10 (CD14^+^CD16^+^CD141^+^CD163^+^) [T(DC-10) cells] or ex vivo cDCs (CD1c^+^CD11c^+^) [T(cDC) cells] FACS-isolated from peripheral blood of healthy donors (ratio 20:1) for 10 days. After culturing, primed CD4^+^ T cells were restimulated with iDCs in vitro differentiated from monocytes from the allogeneic donor used in priming, separately or mixed at a 1:1 ratio. **b**, **c** The percentages of Tr1 cells within T(cDC) and T(DC-10) cell cultures were evaluated by CD49b and LAG-3 expression on CD45RA^−^CD4^+^ T cells (*n* = 10). The following gating strategy was applied: doublet exclusion, followed by alive cells and gating on CD3^+^CD4^+^CD45RA^−^ cells. The percentage of Tr1 cells in each donor tested (**b**) and dot plots from one representative donor tested (**c**) are shown. **d** After 10 days, T cells were restimulated with in vitro differentiated iDCs, which are autologous to cDCs and DC-10 used for priming (*n* = 8), and in some experiments exogenous IL-2 was added to T(DC-10) cell cultures (*n* = 3). Proliferative responses were evaluated by Ki67 staining with flow cytometry. The following gating strategy was applied: doublet exclusion, followed by alive cells and gating on CD3^+^CD4^+^ cells. The percentage of Ki67^+^ T cells in each donor tested (left and middle panel) and histograms from one representative donor tested (right panel) are shown. The red histogram shows the proliferation of T(cDC) cells, and the blue and green histograms correspond to the proliferation of T(DC-10) cells alone and in the presence of IL-2, respectively. The gray shaded histogram shows the isotype control. **e** T(cDC) cells were restimulated with in vitro differentiated iDCs, which are autologous to cDCs and DC-10 used for priming, in the absence or presence of autologous T(DC-10) cells at a 1:1 ratio (*n* = 3). The following gating strategy was applied: doublet exclusion, followed by alive cells and gating on CD3^+^CD4^+^ cells. The percentage of proliferated cells of each donor tested (left panel) and one representative donor (right panel) are shown. The red histogram shows proliferation of T(cDC) cells, while the blue histogram corresponds to proliferation of activated T(cDC) cells in the presence of T(DC-10) cells, and the gray shaded histogram shows unstimulated T(cDC) control. **P ≤ 0.01 ***P ≤ 0.001 (Wilcoxon matched pairs test, two-tailed)

To investigate the induction of alloantigen-specific Tr1 cells, allogeneic naïve CD4^+^ T cells were stimulated for 10 days with ex vivo DC-10 or ex vivo cDCs at a 1:20 ratio. After culturing, CD4^+^ T cells primed with ex vivo DC-10 [T(DC-10) cells] or ex vivo cDCs [T(cDC) cells] were restimulated with cells from the allogeneic donor used in the priming (Fig. 6a). At the end of 10 day culture, the percentage of CD4^+^CD45RA−CD49b^+^LAG-3^+^ cells was assessed as bona fide Tr1 cells.^19^ In all donors tested, CD4^+^ T cells primed with ex vivo DC-10 [T(DC-10) cells] contained a significantly (*P* = 0.002) higher proportion of Tr1 cells compared to T(cDC) cells, with an average of 17.9% and 3.7% of CD49b^+^LAG-3^+^ Tr1 cells, respectively (Fig. 6b, c and S7). Upon secondary stimulation with iDCs from the allogeneic donor used in the priming, T(DC-10) cells were anergic, as demonstrated by the significantly (*P* = 0.0002) lower percentage of Ki67^+^ cells in T(DC-10) cells compared to T(cDC) cell cultures (Fig. 6c, d). Conversely, upon polyclonal stimulation, T(DC-10) cells showed a proliferative response similar to T(cDC) cells (data not shown). The addition of exogenous IL-2 during in vitro alloantigen restimulation restored T(DC-10) cell proliferation (from 32.2 ± 25.9% to 76.6 ± 6.4% of Ki67^+^ cells), similar to that of T(cDC) cells (76 ± 14.15% of Ki67^+^ cells, Fig. 6d). In agreement with the presence of a high frequency of Tr1 cells, T(DC-10) cells efficiently suppressed the proliferation of T(cDC) cells activated with cells from the allogeneic donor used for priming (Fig. 6a, e). These results demonstrate that ex vivo isolated CD14^+^CD16^+^CD141^+^CD163^+^ DC-10 promote alloantigen-specific Tr1 cell differentiation in vitro and are functionally superimposable to in vitro differentiated DC-10.

### The ex vivo DC-10 transcriptome resembles that of monocyte-derived DC-10

**Fig. 7:**
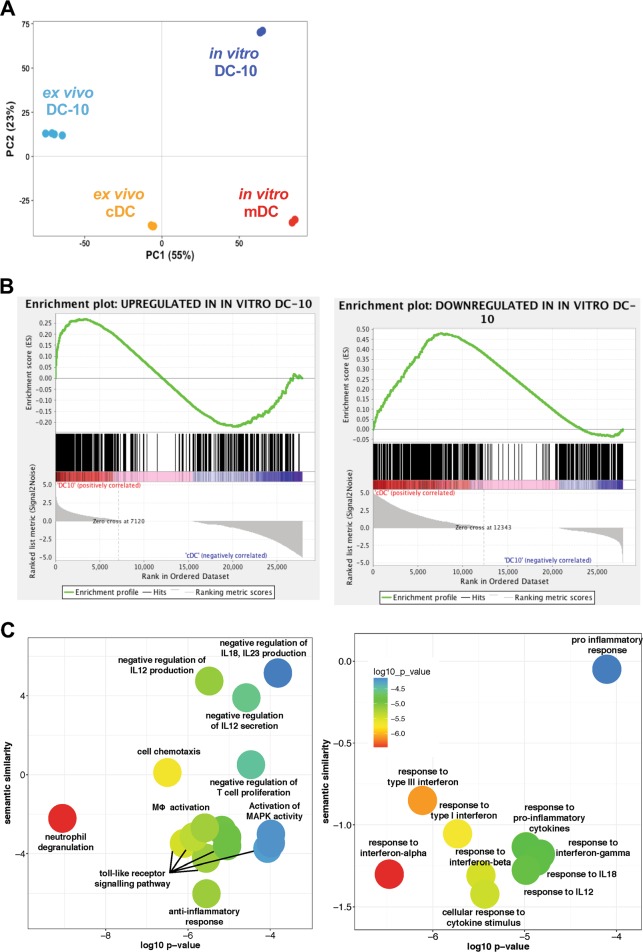
The Ex vivo DC-10 transcriptome resembles that of their in vitro counterpart. RNA was extracted from ex vivo DC-10 (*n* = 4) and from in vitro differentiated DC-10 and mDCs (*n* = 2) and sequenced. Published RNA-seq data of FACS-isolated cDCs (ex vivo cDCs, GSE70106) were used (*n* = 3). **a** Principal component analysis. PC variances are indicated on the axis, and each dot represents a donor and each subset is differently colored. **b** Gene Set Enrichment Analysis in ex vivo DC-10 and cDC transcriptomes of upregulated DEGs (left panel) and downregulated DEGs (right panel) in in vitro differentiated DC-10 compared to mDCs was performed. The enrichment profile is shown by the green line, while the hits are indicated by the black line. **c** The enrichment of biological process GO terms was analyzed with EnrichR and summarized using REVIGO. For each significant class (*P* < 0.01), enriched terms passing the redundancy reduction control are represented as scatterplots. Bubble colors indicate the *P*-value. The Y-axis indicates the semantic similarity between GO terms, whose units have no intrinsic meaning; semantically similar GO terms should cluster together in the plot. Pathways that are upregulated (left panel) and downregulated (right panel) are shown

To study similarities between ex vivo and in vitro differentiated DC-10, we performed RNA-sequencing analyses. As a control, we used donor-matched in vitro differentiated mDCs *(*in vitro mDCs) and published RNA-seq data of FACS-isolated cDCs (ex vivo cDCs, GSE70106^30^). The large majority of transcriptional variance (55%) was linked to the origin of cell populations, with in vitro DC-10 and mDCs divided from ex vivo DC-10 and cDCs by PC1 (Fig. 7a). Interestingly, PC2 (23% of variance) segregated ex vivo and in vitro DC-10 from ex vivo cDCs and in vitro mDCs (Fig. 7a), thus suggesting similarities between ex vivo and in vitro DC-10. High ranking DEGs obtained by in vitro DC-10 versus in vitro mDCs comparison were then used to perform a Gene Set Enrichment Analysis in ex vivo DC-10 and cDC transcriptomes. The top 500 up-regulated genes in in vitro DC-10 were highly expressed by ex vivo DC-10 but not by cDC (Fig. 7b, left panel). Conversely, the top 500 down-regulated genes in in vitro DC-10 were not expressed by ex vivo DC-10, and their expression was significantly upregulated in ex vivo cDCs (enriched at nominal *P-*value <1%; Fig. 7b, right panel).

The Gene Ontology Biological Process (GOBP) enrichment analysis performed on up-regulated and down-regulated genes by both ex vivo isolated and in vitro differentiated DC-10 showed that DC-10 were enriched in genes associated with anti-inflammatory responses as well as negative regulation of T cell proliferation and production of pro-inflammatory cytokines (i.e., IL-12, IL-18, and IL-23). Conversely, responses to type I IFNs and pro-inflammatory cytokines (i.e., IL-12 and IFN-γ), and the pro-inflammatory responses were GOBP-enriched for down-regulated genes. Accordingly, KEGG analysis identified the NF-kB signaling pathway as the most significantly (*P* = 0.006) down-regulated pathway in DC-10 compared to pro-inflammatory DCs (i.e., cDCs and mDCs, data not shown). These pathway analyses highlight the common pro-tolerogenic and anti-inflammatory signatures of ex vivo-isolated and in vitro-differentiated DC-10 cells, demonstrating an overlap between their transcriptomes and confirming that CD14^+^CD16^+^CD141^+^CD163^+^ cells represent the in vivo counterpart of in vitro-generated DC-10.

## Discussion

In this study, we show that the combined expression of CD141 and CD163 with CD14 and CD16 identifies human DC-10. Ex vivo isolated DC-10 from the peripheral blood of healthy donors secrete IL-10 spontaneously at variable levels and consistently upon in vitro activation with limited amounts of IL-12, poorly stimulate allogeneic naïve CD4^+^ T cells, and induce alloantigen-specific anergic Tr1 cells. The co-expression of CD14, CD16, CD141, and CD163 is specific for DC-10 as classical and non-classical monocytes and cDCs do not co-express these markers. The combined expression of CD14, CD16, CD141, and CD163 can be used to isolate and track human DC-10 in vivo.

The expression of CD141 characterizes a population of circulating cDCs, BDCA-3^+^ DCs.^3,31^ Our data show that, although DC-10 and BDCA-3^+^ DCs share CD141 expression, they differ in several aspects: BDCA-3^+^ DCs are lineage-negative cells, while DC-10 express CD14 and CD16; BDCA-3^+^ DCs are TLR4^−^ cells, while we show here that DC-10 respond to LPS, a known TLR4 agonist; BDCA-3^+^ DCs preferentially secrete IL-12,^2^ with an IL-10/IL-12 ratio^32^ opposite to that of DC-10. Moreover, BDCA-3^+^ DCs promote allogeneic CD4^+^ T cell proliferation and IFN-γ secretion, with higher IFN-γ production compared to that induced by CD1c^+^ DCs.^3^ Conversely, DC-10 promote hypo-responsiveness and low IFN-γ production by allogeneic CD4^+^ T cells. Chu et al.^33^ described a population of CD141^+^ DCs in human skin that play an essential role in maintaining skin homeostasis and in regulating systemic and anti-tumor immunity. These DCs share some features with DC-10, including constitutive IL-10 secretion and the ability to induce T cell hypo-responsiveness and Tregs.^33^ It still remains to be defined whether skin-resident CD141^+^ DCs may represent a population of tissue-resident DC-10.

The scavenger receptor CD163 expression is induced by IL-10,^34^ and IL-10 secretion is elicited by CD163 engagement.^35^ CD163 is commonly expressed at high levels in various tissue-resident macrophages polarized towards an M2-like phenotype.^36^ DC-10, similar to M2-like macrophages, express CD163 and up-regulate genes encoding for anti-inflammatory factors. We demonstrate that DC-10 are blood circulating DCs distinct from the described CD163^+^ macrophages or DCs. DC-10 indeed expressed TLR3,^12^ barely expressed CD206 (data not shown), and, once ex vivo isolated from the peripheral blood, prime naïve CD4^+^ T cells in vitro. In contrast to circulating CD163^+^CD11c^+^ DCs,^37^ DC-10 express CD14 and are CD16^bright^. Moreover, DC-10 are CD141^+^CD16^+^, a characteristic that distinguishes them from a recently described population of human intestinal CD14^+^CD163^high^ DCs.^38^ A more in-depth analysis of the presence of DC-10 within the gut mucosa is needed to define whether they may represent a distinct population of DCs that cooperate with other cells to maintain tolerance.

DC-10 have been previously identified in vivo as a subset of CD11c^+^CD14^+^ monocytes expressing CD83.^12^ However, the expression of these markers is not specific for DC-10 because CD14 and CD11c can also be expressed by monocytes and tissue-resident macrophages. Moreover, the expression of CD83 is up-regulated upon cell activation. We now demonstrate that DC-10 can be identified by the concurrent expression of CD14, CD16, CD141, and CD163, and cells isolated with this set of markers display the same cytokine profile and promote anergic T cells in vitro, similar to CD11c^+^CD14^+^CD83^+^ DC-10.^12^ Notably, CD141 and CD163 are expressed by DC-10 regardless of TLR-mediated activation, allowing the discrimination of DC-10 from resting and activated monocytes. And the identification of DC-10 in inflammatory conditions. We show that freshly isolated CD14^+^CD16^+^CD141^+^CD163^+^ cells produce variable levels of IL-10 spontaneously, with some donors secreting high levels of IL-10. We speculate that these variable levels of IL-10 production might be due to environmental stimuli encountered by cells in vivo, which may lead to a different activation cell status. Importantly, upon activation, ex vivo DC-10 produce high amounts of IL-10 in the presence of limited levels of IL-12, and maintain their anti-inflammatory phenotype. Finally, we demonstrate, for the first time, that these ex vivo isolated IL-10-producing DCs promote in vitro anergic alloantigen-specific T cells containing a high proportion of suppressive Tr1 cells, which are features shared with in vitro differentiated DC-10. These data prompted us to conclude that CD14^+^CD16^+^CD141^+^CD163^+^ cells are the tolerogenic DC-10 present in vivo. The similarity between the transcriptional profile of ex vivo isolated and in vitro differentiated DC-10 supports this conclusion. As expected and in agreement with the previously described disparity between ex vivo isolated and in vitro differentiated cells,^39,40^ the transcriptomes of ex vivo and in vitro DC-10 are not completely superimposable. Nevertheless, ex vivo and in vitro DC-10 are transcriptionally distinct from ex vivo cDCs and in vitro mDCs and are characterized by the expression of genes associated with anti-inflammatory responses (i.e., IL-10, CD40LR, FPR2, FPR3, and PTPX3) and down-regulation of pro-inflammatory genes (i.e., type I IFNs, IFN-γ, and TNF responses and CD40), with NF-kB signaling as the most significantly down-regulated pathway. Overall, these data indicate that in vivo occurring DC-10 exhibit an anti-inflammatory and pro-tolerogenic molecular profile, supporting the notion that these cells may play an important role in promoting and/or maintaining tolerance in vivo. It still remains to be defined the origin of ex vivo DC-10, whether these cells are derived from cDC precursors or are monocyte-derived cells. We observed in our RNA-seq analysis that ex vivo DC-10 did not express ZBTB46 (data not shown, see GSE117945). This result is an indirect indication that ex vivo DC-10 are derived from monocytes, as ZBTB46 is specifically expressed by cDCs and committed cDC precursors, but not by monocytes, pDCs, or other immune cell populations.^41^ Future studies will investigate the origin of DC-10 in vivo.

In summary, we discover that the combination of CD14, CD16, CD141, and CD163 allows unambiguous identification of human DC-10 in vivo and represents a significant improvement of the previously described strategy, which relies on the use of the activation-dependent marker CD83. DC-10 isolated with the newly described strategy shows tolerogenic activity, IL-10 secretion and Tr1 cell induction. The definition of this marker combination makes it now feasible to study the role of DC-10 in vivo, which could be crucial for maintaining immunological homeostasis or detrimental in tumor settings.

## Supplementary information

Supplementary Figures

## References

[1] Ziegler-Heitbrock L (2010). Nomenclature of monocytes and dendritic cells in blood. Blood.

[2] Mittag D (2011). Human dendritic cell subsets from spleen and blood are similar in phenotype and function but modified by donor health status. J. Immunol..

[3] Jongbloed SL (2010). Human CD141 + (BDCA-3) + dendritic cells (DCs) represent a unique myeloid DC subset that cross-presents necrotic cell antigens. J. Exp. Med..

[4] Siegal FP (1999). The nature of the principal type 1 interferon – producing cells in human blood. Science.

[5] Moseman EA (2004). Human plasmacytoid dendritic cells activated by CpG oligodeoxynucleotides induce the generation of CD4 + CD25 + regulatory T cells. J. Immunol..

[6] Ito T (2007). Plasmacytoid dendritic cells prime IL-10-producing T regulatory cells by inducible costimulator ligand. J. Exp. Med..

[7] Banchereau J, Steinman RM (1998). Dendritic cells and the control of immunity. Nature.

[8] Iberg CA, Jones A, Hawiger D (2017). Dendritic cells as inducers of peripheral tolerance. Trends Immunol..

[9] Gordon JR, Ma Y, Churchman L, Gordon SA, Dawicki W (2014). Regulatory dendritic cells for immunotherapy in immunologic diseases. Front. Immunol..

[10] Horton C, Shanmugarajah K, Fairchild PJ (2017). Harnessing the properties of dendritic cells in the pursuit of immunological tolerance. Biomed. J..

[11] Waisman Ari, Lukas Dominika, Clausen Björn E., Yogev Nir (2016). Dendritic cells as gatekeepers of tolerance. Seminars in Immunopathology.

[12] Gregori S (2010). Differentiation of type 1 T regulatory cells (Tr1) by tolerogenic DC-10 requires the IL-10-dependent ILT4/HLA-G pathway. Blood.

[13] Amodio G (2015). HLA-G expression levels influence the tolerogenic activity of human DC-10. Haematologica.

[14] Bacchetta R (2010). Molecular and functional characterization of allogantigen-specific anergic T cells suitable for cell therapy. Haematologica.

[15] Petrelli A (2015). Generation of donor-specific t regulatory type 1 cells from patients on dialysis for cell therapy after kidney transplantation. Transplantation.

[16] Comi M, Amodio G, Gregori S (2018). Interleukin-10-producing DC-10 Is a unique tool to promote tolerance via antigen-specific T regulatory type 1 cells. Front. Immunol..

[17] Amodio G (2013). HLA-G expressing DC-10 and CD4(+) T cells accumulate in human decidua during pregnancy. Hum. Immunol..

[18] Repnik U, Knezevic M, Jeras M (2003). Simple and cost-effective isolation of monocytes from buffy coats. J. Immunol. Methods.

[19] Gagliani N (2013). Coexpression of CD49b and LAG-3 identifies human and mouse T regulatory type 1 cells. Nat. Med..

[20] Gautier L, Cope L, Bolstad BM, Irizarry RA (2004). Affy - analysis of Affymetrix GeneChip data at the probe level. Bioinformatics.

[21] Ritchie ME (2015). Limma powers differential expression analyses for RNA-sequencing and microarray studies. Nucleic Acids Res..

[22] Jiang H., Lei R., Ding S. W., Zhu S. Skewer: A fast and accurate adapter trimmer for next-generation sequencing paired-end reads. *BMC Bioinform.* 2014; 1**5**. 10.1186/1471-2105-15-182. 10.1186/1471-2105-15-182 PMC4074385 24925680

[23] Dobin A (2013). STAR: Ultrafast universal RNA-seq aligner. Bioinformatics.

[24] Love M. I., Huber W., Anders S. Moderated estimation of fold change and dispersion for RNA-seq data with DESeq2. *Genome Biol.* 2014; 15. 10.1186/s13059-014-0550-8. 10.1186/s13059-014-0550-8 PMC4302049 25516281

[25] Subramanian A (2005). Gene set enrichment analysis: a knowledge-based approach for interpreting genome- wide expression profiles gene set enrichment analysis: a knowledge-based approach for interpreting genome-wide expression profiles. Proc. Natl Acad. Sci. USA.

[26] Chen Edward Y, Tan Christopher M, Kou Yan, Duan Qiaonan, Wang Zichen, Meirelles Gabriela, Clark Neil R, Ma’ayan Avi (2013). Enrichr: interactive and collaborative HTML5 gene list enrichment analysis tool. BMC Bioinformatics.

[27] Supek Fran, Bošnjak Matko, Škunca Nives, Šmuc Tomislav (2011). REVIGO Summarizes and Visualizes Long Lists of Gene Ontology Terms. PLoS ONE.

[28] Mahnke K, Schmitt E, Bonifaz L, Enk AH, Jonuleit H (2002). Immature, but not inactive: the tolerogenic function of immature dendritic cells. Immunol. Cell Biol..

[29] Levings MK (2005). Differentiation of Tr1 cells by immature dendritic cells requires IL-10 but not CD25 + CD4 + Tr cells. Blood.

[30] Kumar NA (2015). The role of antigen presenting cells in the induction of HIV-1 latency in resting CD4(+) T-cells. Retrovirology.

[31] Macdonald KPa (2002). Characterization of human blood dendritic cell subsets. Blood.

[32] Gupta MR, Kolli D, Garofalo RP (2013). Differential response of BDCA-1+ and BDCA-3+ myeloid dendritic cells to respiratory syncytial virus infection. Respir. Res..

[33] Chu CC (2012). Resident CD141 (BDCA3)+ dendritic cells in human skin produce IL-10 and induce regulatory T cells that suppress skin inflammation. J. Exp. Med..

[34] Williams L, Jarai G, Smith A, Finan P (2002). IL-10 expression profiling in human monocytes. J. Leukoc. Biol..

[35] Philippidis P (2004). Hemoglobin scavenger receptor CD163 mediates interleukin-10 release and heme oxygenase-1 synthesis: antiinflammatory monocyte-macrophage responses in vitro, in resolving skin blisters in vivo, and after cardiopulmonary bypass surgery. Circ. Res..

[36] Mosser DM (2003). The many faces of macrophage activation. J. Leukoc. Biol..

[37] Maniecki MB, Møller HJ, Moestrup SK, Møller BK (2006). CD163 positive subsets of blood dendritic cells: The scavenging macrophage receptors CD163 and CD91 are coexpressed on human dendritic cells and monocytes. Immunobiology.

[38] Barman S (2016). Identification of a human intestinal myeloid cell subset that regulates gut homeostasis. Int. Immunol..

[39] Balan S (2014). Human XCR1 ^+^ dendritic cells derived in vitro from CD34 ^+^ progenitors closely resemble blood dendritic cells, including their adjuvant responsiveness, contrary to monocyte-derived dendritic cells. J. Immunol..

[40] Alcántara-Hernández M (2017). High-dimensional phenotypic mapping of human dendritic cells reveals interindividual variation and tissue specialization. Immunity.

[41] Meredith MM (2012). Expression of the zinc finger transcription factor zDC (Zbtb46, Btbd4) defines the classical dendritic cell lineage. J. Exp. Med..

