# Supplementary material for: Coexpression of CD163 and CD141 identifies human circulating IL-10-producing dendritic cells (DC-10)
Source: Cell Mol Immunol. 2019 Mar 6;17(1):95–107. doi: 10.1038/s41423-019-0218-0 (PMC6952411; doi:10.1038/s41423-019-0218-0)
Supplement: Supplementary file 1 — Supplementary Figures [file 41423_2019_218_MOESM1_ESM.pdf]

Co-expression of CD163 and CD141 Identifies Human Circulating IL-10-Producing Dendritic Cells (DC-10)

Michela Comi[1,2], Daniele Avancini[1], Francesca Santoni de Sio[1], Matteo Villa[1], Molly Javier Uyeda[3], Matteo Floris[4], Daniela Tomasoni[1], Alessandro Bulfone[4], Maria Grazia Roncarolo[3*], and Silvia Gregori[1*]

Running title: *In vivo* occurring tolerogenic DC-10

[1]San Raffaele Telethon Institute for Gene Therapy (SR-TIGET), San Raffaele Scientific Institute (IRCCS) Milan, Italy; [2]PhD Program in Translational and Molecular Medicine (DIMET), University of Milan-Bicocca, Italy; [3]Division of Stem Cell Transplantation and Regenerative Medicine, Department of Pediatrics, ISCBRM, Stanford School of Medicine, Stanford, California, USA; [4]CRS4, Technology Park Polaris, Pula, Italy;

*These two authors contributed equally to this work

Corresponding author:
Silvia Gregori,
San Raffaele Telethon Institute for Gene Therapy (SR-TIGET),
IRCCS San Raffaele Scientific Institute.
Via Olgettina, 58, 20132 Milan, Italy.
Phone: +39 0226434894;
Fax: +39 0226434668;
E-mail: gregori.silvia@hsr.it

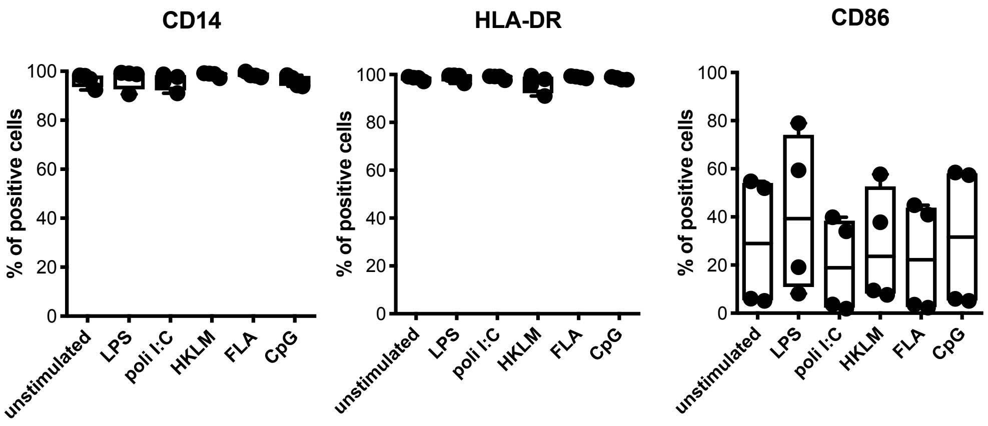

Comi M. et al., Figure S1

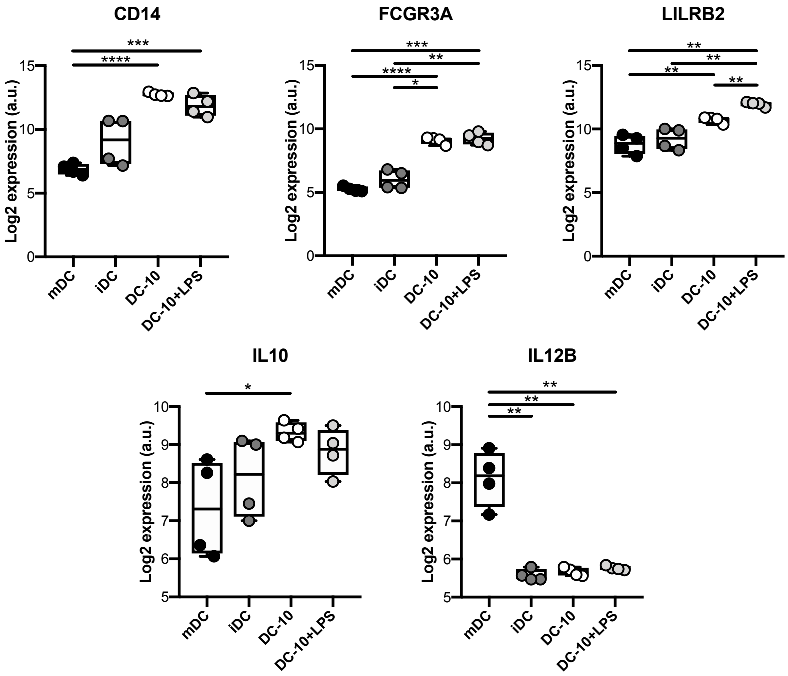

Comi M. et al., Figure S2

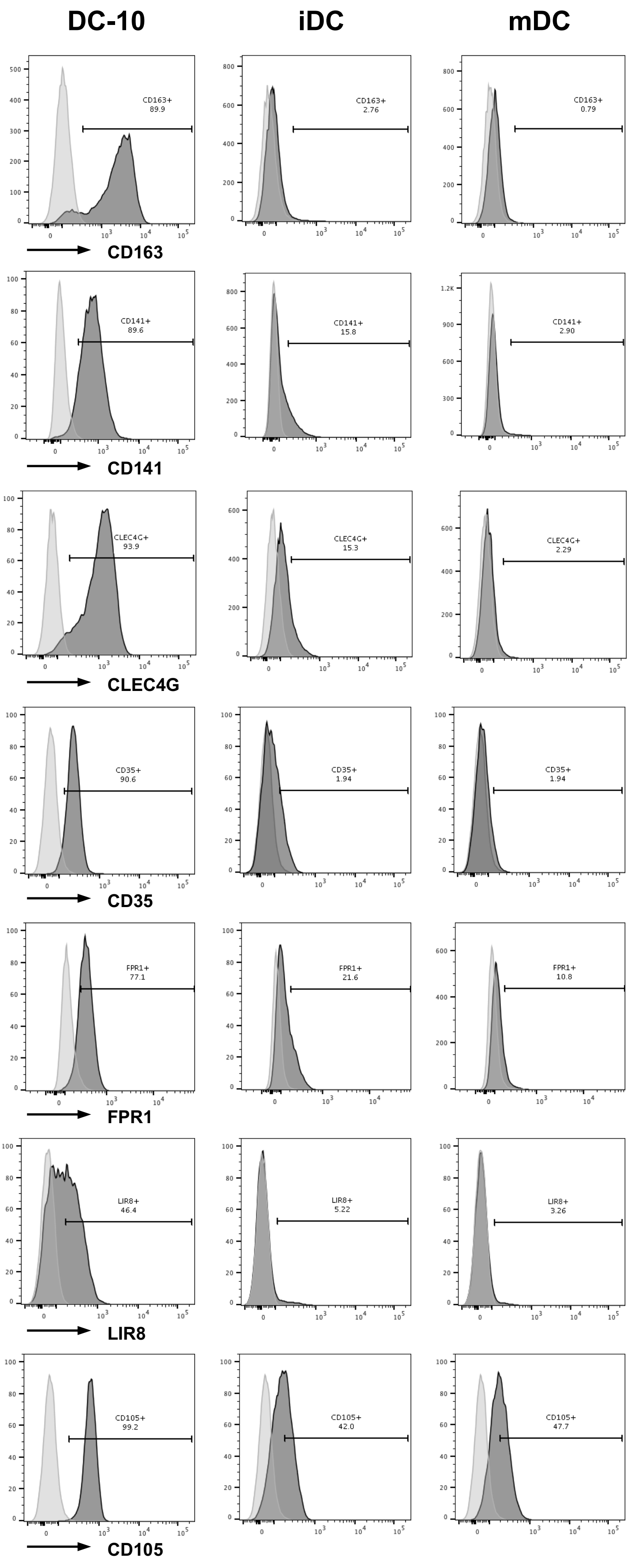

Comi M. et al., Figure S3

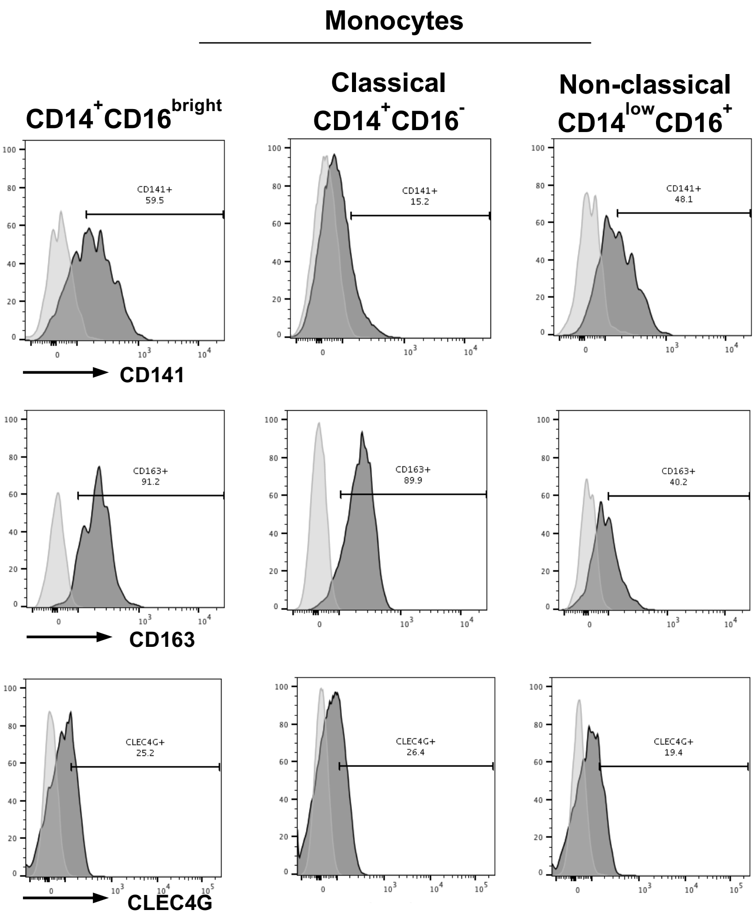

Comi M. et al., Figure S4

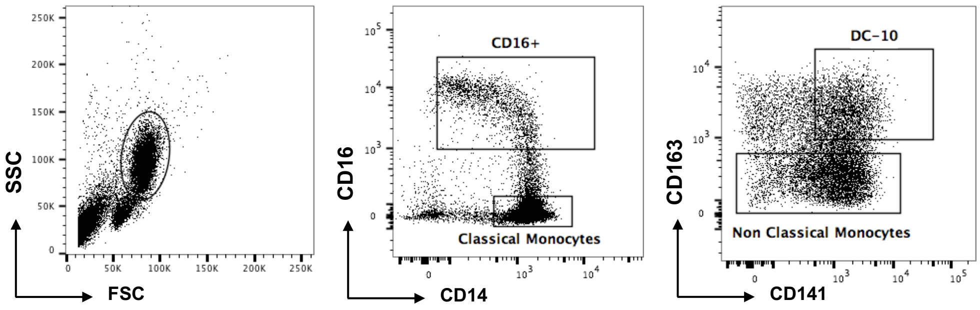

Comi M. et al., Figure S5

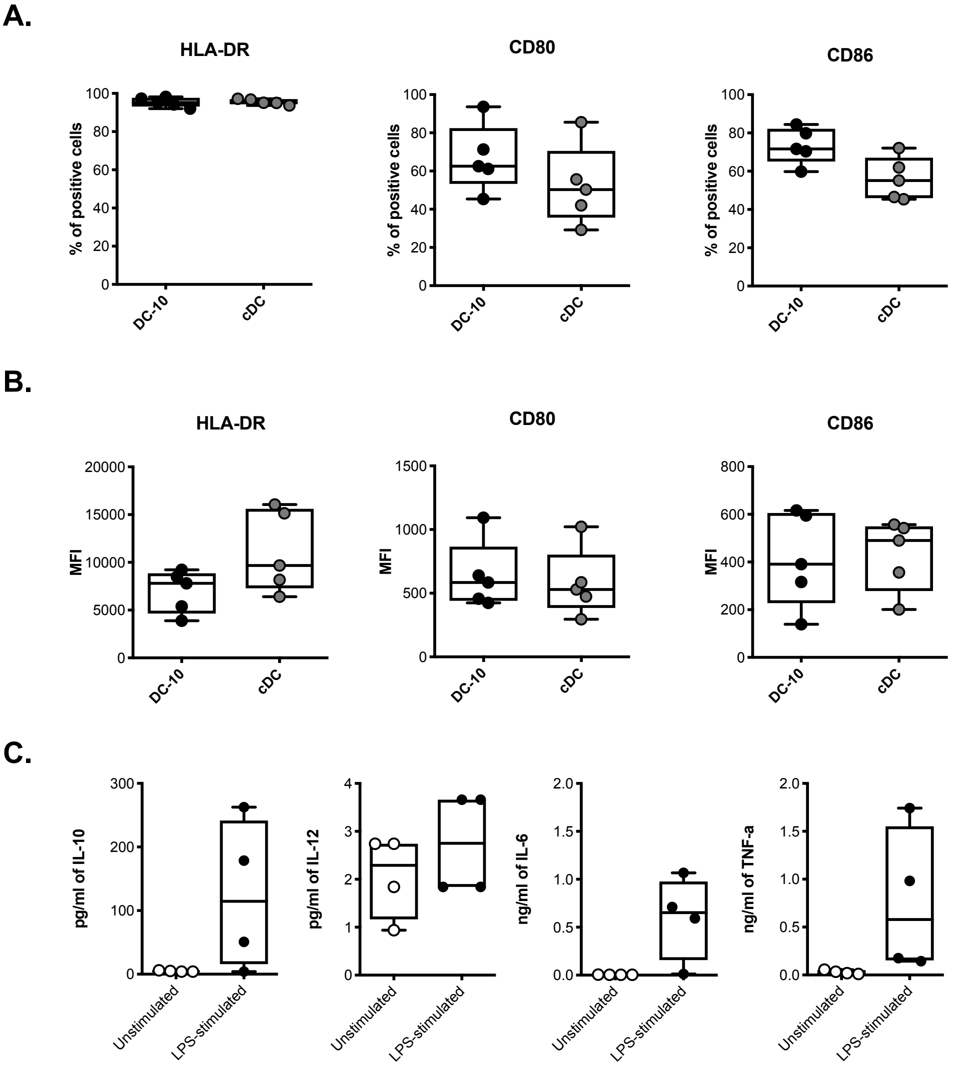

Comi M. et al., Figure S6

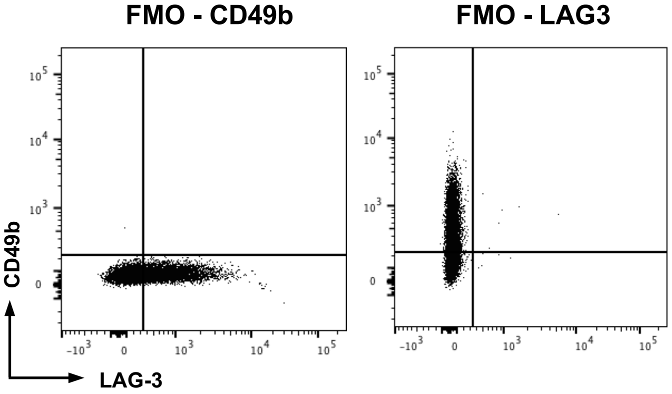

Comi M. et al., Figure S7

Supplementary Figure legends

Figure S1. Stability of DC-10 phenotype upon TLR stimulation. DC-10 were differentiated *in vitro* from peripheral blood monocytes according to material and methods and activated at day 5 with the indicated TLR2/3/5/9 agonists. Expression of CD14, HLA-DR and CD86 evaluated by flow cytometry is shown. The following gating strategy was applied: doublets, followed by alive cells, and gating on $CD11c^{+}$ cells. Each dot represents a single donor, lines indicate median, while whiskers are minimum and maximum levels.

Figure S2. Validation of genes encoding for DC-10-associated biomarkers. Mature (m)DC, immature (i)DC, DC-10 and DC-10 activated with LPS (DC-10+LPS) were differentiated *in vitro* from peripheral blood monocytes of healthy donors (n=4) according to methods and microarray analysis was performed. Expression of *CD14*, *FCGR3A*, *LILRB2*, *IL-10* and *IL12B* determined by DNA microarray is shown. Each dot represents a single donor, lines indicate median, while whiskers are minimum and maximum levels. *P≤0.05, **P≤0.01, ***P≤0.001, ****P≤0.0001.

Figure S3. Fluorescence Minus One (FMO) control staining of putative DC-10 markers on *in vitro* differentiated DC. Mature (m)DC, immature (i)DC, and DC-10 were differentiated *in vitro* from peripheral blood monocytes of healthy donors according to material and methods and stained with antibodies against the indicated markers. The following gating strategy was applied: doublet exclusion, followed by alive cells, and gating on $CD11c^{+}$ cells. One representative donor (shaded black) with the corresponding FMO staining (shaded grey) is presented.

Figure S4. Fluorescence Minus One (FMO) control staining of putative DC-10 markers on *in vivo* peripheral $CD14^{+}$ cells. $CD14^{+}CD16^{+}$ cells, $CD14^{+}CD16^{-}$ (classical), and $CD14^{low}CD16^{+}$ (non-classical) monocytes in peripheral blood were analysed for CD141, CD163 and CLEC4G expression. One representative donor (shaded black) with the corresponding FMO staining (shaded grey) is presented. The following gating strategy was applied: doublet exclusion, followed by PBMC, and gating on $CD14^{dim}CD16^{+}$ (non-classical monocytes), $CD14^{bright}CD16^{+}$ and $CD14^{+}CD16^{-}$ (classical monocytes) cells.

Figure S5. Gating strategy for *ex vivo* DC-10 isolation. DC-10 were identified in the peripheral blood according to FSC^{high}/SSC^{high} physical parameters and CD14, CD16, CD141 and CD163 co-expression. Classical monocytes were isolated as $CD14^{+}CD16^{-}$, while non-classical monocytes were $CD163^{-}$ cells within $CD16^{+}$ gate. Dot plots from one representative donor are shown.

Figure S6. Phenotype and cytokine profile of *ex vivo* cDC. A-B. Expression of HLA-DR, CD80 and CD86 was evaluated on *ex vivo* DC-10 ($CD14^{+}CD16^{+}CD141^{+}CD163^{+}$ cells), *ex vivo* cDC (n=4).

Percentage of positive cells (**A**) and Mean Fluorescence Intensity (MFI; **B**) is presented. Each dot represents a single donor, lines indicate median, while whiskers are minimum and maximum levels. The following gating strategy was applied: doublet exclusion, followed by PBMC, and gating on $CD14^+CD16^+CD141^+CD163^+$ (*ex vivo* DC-10) and $CD11c^+CD1c^+$ (*ex vivo* cDC) cells. **C.** *Ex vivo* cDC were left unstimulated or stimulated with LPS for 48 hours. Concentration levels of IL-10, IL-6, IL-12, and TNF-α in culture supernatants were evaluated by multiplex microbead-based cytokine array. Each dot represents a single donor, lines indicate median, while whiskers are minimum and maximum levels.

Figure S7. Fluorescence Minus One (FMO) control staining of CD49b and LAG-3 on $CD4^+$ T cells. Naive $CD4^+$ T cells were cultured with *ex vivo* DC-10 or *ex vivo* cDC FACS-isolated from peripheral blood of healthy donors (ratio 20:1) for 10 days. FMO staining for CD49b (left) and LAG-3 (right) of one representative donor is shown. The following gating strategy was applied: doublet exclusion, followed by alive cells and gating on $CD3^+CD4^+CD45RA^-$ cells.
